# Contrasting effects of the α7 nicotinic receptor antagonist methyllycaconitine in different rat models of heroin reinstatement

**DOI:** 10.1177/0269881121991570

**Published:** 2021-03-10

**Authors:** Josephine Palandri, Sharon L Smith, David J Heal, Sue Wonnacott, Chris P Bailey

**Affiliations:** 1 Department of Pharmacy and Pharmacology, University of Bath, Bath, UK; 2 RenaSci Ltd, BioCity, Nottingham, UK; 3 DevelRx Ltd, BioCity, Nottingham, UK; 4 Department of Biology and Biochemistry, University of Bath, Bath, UK

**Keywords:** Heroin, opioid, conditioned place preference, intravenous self-administration, drug-seeking, reinstatement, relapse, abuse

## Abstract

**Background:**

α7 Nicotinic acetylcholine receptors are implicated in the reinstatement of drug-seeking, an important component of relapse. We showed previously that the α7 nicotinic acetylcholine receptor antagonist, methyllycaconitine, specifically attenuated morphine-primed reinstatement of conditioned place preference in rodents and this effect was mediated in the ventral hippocampus.

**Aims:**

The purpose of this study was to evaluate α7 nicotinic acetylcholine receptor antagonism in reinstatement of the conditioned place preference for the more widely abused opioid, heroin, and to compare the effect of α7 nicotinic acetylcholine receptor blockade on reinstatement of heroin-seeking and heroin self-administration in an intravenous self-administration model of addictive behaviour.

**Methods:**

Rats were trained to acquire heroin conditioned place preference or heroin self-administration; both followed by extinction of responding. Methyllycaconitine or saline was given prior to reinstatement of drug-primed conditioned place preference, or drug-prime plus cue-induced reinstatement of intravenous self-administration, using two protocols: without delivery of heroin in response to lever pressing to model heroin-seeking, or with heroin self-administration, using fixed and progressive ratio reward schedules, to model relapse.

**Results:**

Methyllycaconitine had no effect on acquisition of heroin conditioned place preference or lever-pressing for food rewards. Methyllycaconitine blocked reinstatement of heroin-primed conditioned place preference. Methyllycaconitine did not prevent drug-prime plus cue-induced reinstatement of heroin-seeking, reinstatement of heroin self-administration, or diminish the reinforcing effect of heroin.

**Conclusions:**

The α7 nicotinic acetylcholine receptor antagonist, methyllycaconitine, prevented reinstatement of the opioid conditioned place preference, consistent with a role for α7 nicotinic acetylcholine receptors in the retrieval of associative memories of drug liking. The lack of effect of methyllycaconitine in heroin-dependent rats in two intravenous self-administration models suggests that α7 nicotinic acetylcholine receptors do not play a role in later stages of heroin abuse.

## Introduction

Drug addiction is defined as a chronic relapsing disorder (Koob, 2020; Koob and Volkow, 2016). Particularly noteworthy is the current escalation of addiction to opioid drugs worldwide. This has been attributed to over-prescription of painkillers and increased availability of illicit synthetic opioids: heroin and the vastly more dangerous fentanyl (Pergolizzi et al., 2018). In the USA the ‘opioid epidemic’ resulted in 2017 in 68% of all drug overdose deaths, with over 47,000 being related to opioids (Scholl et al., 2018).

Relapse not only sustains the addiction cycle (Koob and Volkow, 2016) but also poses a particular challenge for addiction therapies. For opioids, relapse rates to first reuse are approximately 60% after 3 months and 80% after 12 months of abstinence (Bart, 2012; Dong et al., 2017). Major triggers for relapse are stress, drug-associated cues and re-exposure to drugs (Perry et al., 2014; Shalev et al., 2002), and susceptibility persists despite long periods of abstinence (Bart, 2012). Understanding the molecular mechanisms underlying the relapse in opioid addiction will inform the development of more efficacious treatments.

Opioids induce strong neuroadaptations associated with the key characteristics of dependence, including tolerance, withdrawal and contextual and emotional associations that contribute to compulsive use, cravings and relapse (Chartoff and Connery, 2014; Dong et al., 2017; Koob, 2020; Madsen et al., 2012).A complex circuitry involving many neurotransmitters and brain regions underpins the incentive salience of rewarding drugs (Koob and Volkow, 2016). These mechanisms differ between drug classes, reflecting the diverse neurochemical targets and their cellular functions, for example the dominant role of dopamine in psychostimulant abuse is less compelling for opioids (Badiani et al., 2011). However, reinstatement of drug-seeking involves the re-establishment of goal-directed behaviours, mediated, in large part, by interconnections between a number of brain regions that are common to reinstatement across all drugs of abuse (prefrontal cortex, hippocampus, nucleus accumbens, amygdala, ventral tegmental area (Koob and Volkow, 2016; Namba et al., 2018)). There are differences in how initiators of the reinstatement of drug-seeking, e.g. contextual cues, drug priming, stress and combinations thereof, engage with this circuitry (Badiani et al., 2011; Keogh et al., 2017; Namba et al., 2018; Rogers et al., 2008) and these can vary in their relative contributions across drug classes.

Cholinergic inputs to the reward circuitry, from the basal forebrain and pedunculopontine and laterodorsal tegmental nuclei, are associated with arousal, attention, learning and reward. Of particular interest are nicotinic acetylcholine receptors (nAChRs) that not only mediate actions of endogenous acetylcholine but also the effects of exogenous nicotine, and are responsible for sustaining tobacco addiction (Wonnacott et al., 2005). nAChRs contribute to relevant cognitive behaviours, including attention (notably to cues that elicit goal-oriented behaviours, Sarter, 2015), motivation, learning and memory (Kutlu and Gould, 2016). In addition, nAChRs have been implicated in the reinforcing behaviours of drugs of abuse other than nicotine (Bajic et al., 2015; Feduccia et al., 2012; Neugebauer et al., 2013), and have been proposed as novel targets for treating addiction (Rahman et al., 2015).

nAChRs are heterogeneous with respect to subunit composition, cellular localisation and regional distribution, properties and physiological roles (Zoli et al., 2015). While heteropentameric nAChR subtypes are the major players in nicotine dependence and have been implicated in other addictions (Walters et al., 2006; Neugebauer et al., 2013; Picciotto and Kenny, 2013), homopentameric α7 nAChRs have a role in the reinstatement of drug-seeking behaviours (Feng et al., 2011; Liu, 2014; Wright et al., 2019). Feng et al. (2011) reported that the selective α7 nAChR antagonist methyllycaconitine (MLA) blocked the reinstatement of morphine-primed conditioned place preference (CPP) in mice. We repeated and extended this observation, showing that MLA had no effect on the acquisition, expression, maintenance or reconsolidation of morphine CPP in mice, but attenuated the reinstatement of morphine-primed CPP in mice and rats (Wright et al., 2019). Furthermore, systemic MLA inhibited morphine-primed increases in ^3^H-AMPA (α-amino-3-hydroxy-5-methyl-4-isoxazolepropionic acid) receptor binding in the ventral hippocampus, and MLA infused directly into this brain area (but not the dorsal hippocampus or prefrontal cortex) abolished morphine-primed reinstatement of CPP (Wright et al., 2019). These results suggest a role for α7 nAChRs in reinstatement of opiate-liking.

The present study aimed to examine the effects of MLA on the reinforcing properties of heroin and reinstatement of heroin-seeking and heroin-taking. In terms of abuse liability, heroin is a more potent and socially relevant opioid than morphine. Reinstatement in two different models of reward-based learning was compared, to interrogate different aspects of relapse. The reinstatement of CPP provides a model relevant to the acquisition of drug liking. Intravenous self-administration (IVSA) provides a representative model of volitional drug taking in psychologically dependent animals and was used in two configurations to model reinstatement of drug-seeking (craving) and reinstatement of drug self-administration (abuse). We observed different effects of α7 nAChR inhibition by MLA in these models: drug seeking in heroin-primed CPP was abolished by MLA, whereas reinstatement of drug seeking or drug self-administration was unaffected by MLA.

## Materials and methods

### Animals

All experiments were approved by the UK Home Office and performed in accordance with the UK Animals (Scientific Procedures) Act of 1986 and conformed to the Animals Research: Reporting In Vivo Experiments (ARRIVE) guidelines (Kilkenny et al., 2010). All animals were handled and weighed daily (except at weekends) for 1 week prior to the start of experiments.

#### CPP

Male Wistar rats (5–9 weeks of age at the start of experiments; University of Bath breeding colony) were housed in groups of four in a controlled environment [12:12 h light-dark cycle (lights on 07:00), constant temperature (24 ± 2°C) and humidity (55 ± 5%)]. Food and water were available *ad libitum*. All experiments were conducted during the light phase, between 08:00–18:00 under dim white light (approximately 15 lux light intensity).

#### IVSA

Male Sprague Dawley rats (approx. 200–225 g on arrival; Charles River, Kent, UK) were single-housed in the same room as the operant chambers in a controlled environment [12:12 h light-dark cycle (lights on 07:00), constant temperature (24 ± 2°C) and humidity (55 ± 20%)], with unlimited access to water. Rats had a daily socialisation session in a large arena to mitigate against social isolation stress. Rats were placed on a 90% food restriction regimen except during the first week of habituation, 24 h pre-surgery and 48 h post-surgery, during which time food was available *ad libitum*. All training and experimental sessions were conducted during the light phase.

Although different strains were used in the two behavioural paradigms based on local precedent, Wistar rats perform well in heroin self-administration (Carrera et al., 1999), and Sprague Dawley rats in heroin CPP experiments (Turner et al., 2014), suggesting no confounding effects of strain on the results in this report.

### Drugs

Heroin hydrochloride was purchased from MacFarlan Smith, Edinburgh, UK; MLA was purchased from Abcam, Cambridge, UK. MLA was dissolved in sterile saline (0.9% w/v) and injected subcutaneously (s.c.) at a volume of 1 mL/kg. Control animals received the same volume of vehicle injections (saline 0.9% w/v, Hameln pharmaceuticals, Gloucester, UK) s.c. Heroin was dissolved in saline (0.9% w/v). Heroin and saline for intravenous (i.v.) infusion were filter-sterilised through a 0.22 µm filter and were administered at 0.5 mL/kg.

### CPP procedure (performed at University of Bath)

CPP was carried out as previously described (Cordery et al., 2014; Wright et al., 2019) in three-compartment shuttle boxes (Tracksys, Nottingham, UK). Compartments were 40x40 cm, linked by a 10x20 cm central area. One compartment consisted of sides with horizontal black and white stripes and a metal floor with circular holes, the other consisted of sides with vertical black and white stripes and a metal floor with square holes. During all preference test sessions (typically 15 min), animals had free access to both compartments, and the time spent in each compartment and their locomotor activity (distance travelled) were recorded using EthoVision XT version 8.0 (Tracksys) tracking software. ‘Preference score’ was taken as [time spent in drug-paired side (s)] − [450 (s)], i.e. half the maximum time, to present the data as a change in preference from 0 (neutral).

#### Acquisition of heroin CPP

**Figure 1.**
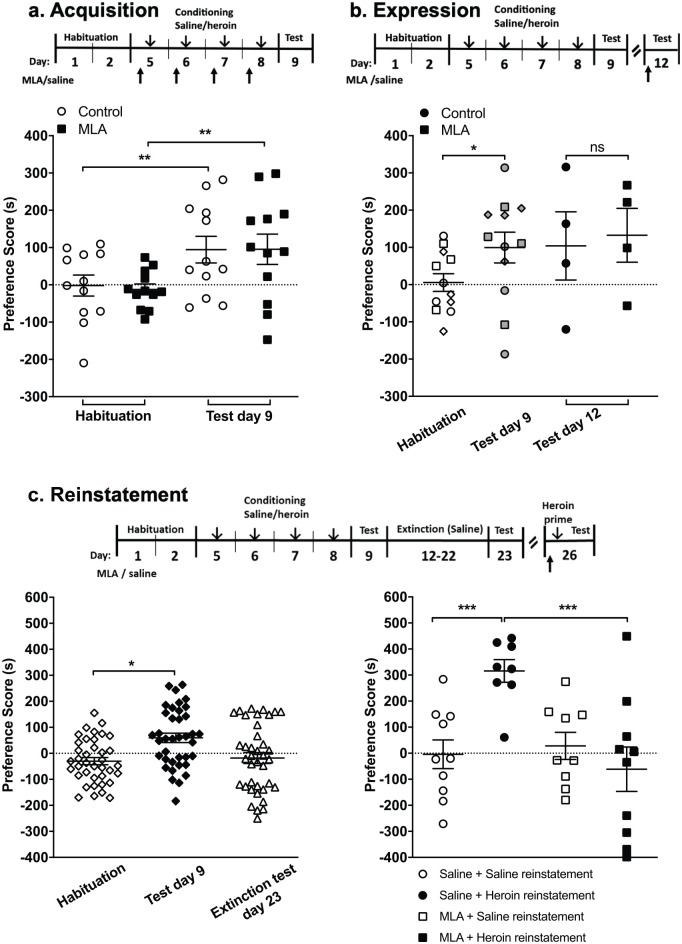
Effect of methyllycaconitine (MLA) on the stages of heroin conditioned place preference (CPP) in male Wistar rats. (a) Acquisition: rats were tested for innate preference (habituation), and then pseudo-randomly assigned to two groups with comparable mean preference scores. The MLA treatment group received MLA (◻, 4 mg/kg, s.c.) 20 min prior to a conditioning dose of heroin (1 mg/kg, s.c.), paired with the drug-paired compartment, and prior to saline, in the unpaired compartment, alternating over four consecutive days. The control group received saline (○, 1 mL/kg, s.c.) instead of MLA. A post-conditioning preference test was conducted the day after the last conditioning session (test day 9), in which rats had free access to the CPP apparatus compartments for 15 min. Preference scores indicate the time spent in the heroin-paired compartment in seconds minus 450 (half the total time). **p < 0.01, two-way analysis of variance (ANOVA) with Bonferroni post-hoc test, *n* = 12 per treatment group. (b) Expression: 12 rats (◊, ○, □) were conditioned to acquire heroin CPP as in (a) and were tested for preference for the drug paired side (test day 9). Rats marked ◊ were sacrificed prior to further testing. Rats marked ○ and □ in the habituation and test day 9 were subsequently administered either saline (•) or MLA (■) in the expression test (test day 12). Three days after the post conditioning test, rats were pseudo-randomly assigned to a treatment group and given either saline (•) or MLA (◻, 4 mg/kg, s.c.) 20 min prior to an additional 15-minute preference test (test day 12). *p < 0.05, habituation vs test day 9, *n* = 12 (paired *t*-test); *n* = 4 per treatment group for expression (test day 12; paired *t*-test). (c) Reinstatement: left: rats were tested for initial preference (◊, habituation), then conditioned to acquire heroin CPP as in (a), followed by 9 days of extinction (saline injections only, paired with alternate compartments on different days). All rats acquired heroin CPP (♦, test day 9) and showed no significant preference in the post-extinction test (∆, test day 23, *p < 0.05, two-way ANOVA with Bonferroni post-hoc analysis vs habituation, *n* = 37). Right: on the reinstatement test day 26, rats were assigned to one of four treatment groups with comparable mean preference scores from habituation (◊), post-conditioning (♦, test day 9) and post-extinction (∆, test day 23): saline control (○, Saline+Saline, *n* = 10), heroin reinstatement (•, Saline+Heroin, *n* = 8), MLA control (□, MLA+Saline, *n* = 9) or MLA reinstatement (◻, MLA+Heroin, *n* = 10), where MLA (4 mg/kg, s.c.) or saline was administered 20 min prior to a priming dose of heroin (1 mg/kg, s.c.) or saline (1 mL/kg, s.c.). Rats were then placed free-roaming in an extended preference test (30 min); data from the second 15 min bin are presented. ***p < 0.005, two-way ANOVA with Bonferroni post-hoc analysis vs heroin reinstatement. Data are expressed as mean ± standard error of the mean (SEM).

Rats underwent two drug-free habituation trials (1 × 15 min session/day for 2 consecutive days) to detect any innate preference for either compartment. Animals were then pseudo-randomly assigned to treatment groups so that mean baseline preferences were close to zero (Figure 1). Heroin conditioning consisted of 1 × 40 min trial/day for 4 days, starting 2 days after the habituation test. To determine the effect of α7 nAChR antagonism on the acquisition of heroin CPP, animals were administered MLA (4 mg/kg, s.c.) or saline in the home cage, 20 min before receiving a conditioning dose of heroin (1 mg/kg, s.c.) or saline. The rats were confined to the drug-paired or saline-paired compartment respectively. The drug/saline pairing and compartment were reversed on consecutive days so that each animal received two heroin injections and two saline injections. A counterbalanced design was employed so that within a treatment group, half the rats were drug-paired with one compartment type and the other half were drug-paired with the other compartment type; and the order of heroin or saline administration was also counterbalanced.

#### Expression of heroin CPP

Animals (*n* = 12) acquired heroin CPP as above and underwent a second post-conditioning preference text; 3 days later, animals received either MLA (4 mg/kg, s.c., *n* = 4) or saline (*n* = 4) 20 min before an additional 15-minute post-conditioning preference test.

#### Extinction and reinstatement of heroin CPP

Animals acquired heroin CPP (*n* = 48), as described previously (Figure 1(a) and (b)), followed by extinction training. Animals received saline injections only, paired with alternate compartments of the CPP box, over 9 consecutive days. On the following day, the animals were subjected to another preference test (*n* = 12 per treatment group). Three days later, for the reinstatement of heroin CPP, animals received a priming dose of heroin (1 mg/kg s.c.) prior to preference testing. This priming dose of heroin was previously shown to induce reinstatement of CPP in rats (Leri and Rizos, 2005; Tzschentke, 2007; Van Der Kam et al., 2009). An extended preference test (30 min based on our previous findings (Wright et al., 2019)) was given, with free access to both compartments of the CPP box; the time spent in each compartment was monitored over two consecutive 15 min time-bins. Preliminary data showed development of reinstatement over the 30-minute test session (as reported in Mueller et al., 2002), therefore, the second 15 min time-bin was taken for reinstatement values. MLA (4 mg/kg s.c.) or saline was administered 20 min before the priming dose of heroin.

#### Statistical analysis

Animals were excluded from statistical analysis if extinction training failed to meet criterion (less than 70% of time spent in the previously drug-paired compartment) – 11 of 48 rats; or if a statistical outlier (Grubb’s outlier test) – one animal was excluded.

The effect of MLA on the acquisition and reinstatement of heroin CPP was analysed by two-way analysis of variance (ANOVA) with Bonferroni post-hoc analysis. The effect of MLA on the expression of heroin CPP was analysed by Student’s *t*-test.

## Heroin IVSA procedure (performed at RenaSci)

IVSA was carried out essentially as previously described (Smith et al., 2019). All experimental sessions were conducted in operant chambers (30.5 × 24.1 × 21.0 cm), located inside sound-attenuating, ventilated cubicles (Med Associates, Inc., St Albans, Vermont, USA). Each chamber was equipped with two levers located 11.5 cm apart with a 5 × 5 cm opening between the levers for food pellet delivery from a food hopper. Data were collected and stored by a computer system and associated interface (Med Associates, Inc.). Each sound attenuating cubicle was equipped with an infra-red camera (RF Concepts or Med Associates, Inc.) from which images were relayed to a computer monitor. Drug or saline was delivered using PHM-100A single speed syringe pumps (Med Associates, Inc.).

### Food training

After 1 week of habituation, rats (*n* = 26) were trained to lever-press for food pellets (45 mg dustless precision pellets, Bilaney Consultants Ltd.) on a fixed ratio (FR) 1 schedule. Training sessions lasted for 1 h or until a rat had received 50 food pellet rewards. Once rats had acquired lever-pressing on the FR1 schedule, the response requirement was increased to FR2, then FR3 and the left lever was assigned as the active lever. Thereafter, only responses on the left lever resulted in the delivery of a reward. Operant responding was deemed stable under the FR3 schedule when the rats earned ⩾45 food pellets within 1 h over four consecutive sessions. All rats acquired FR3 lever-pressing.

### Effect of MLA on operant responding for food rewards

**Figure 2.**
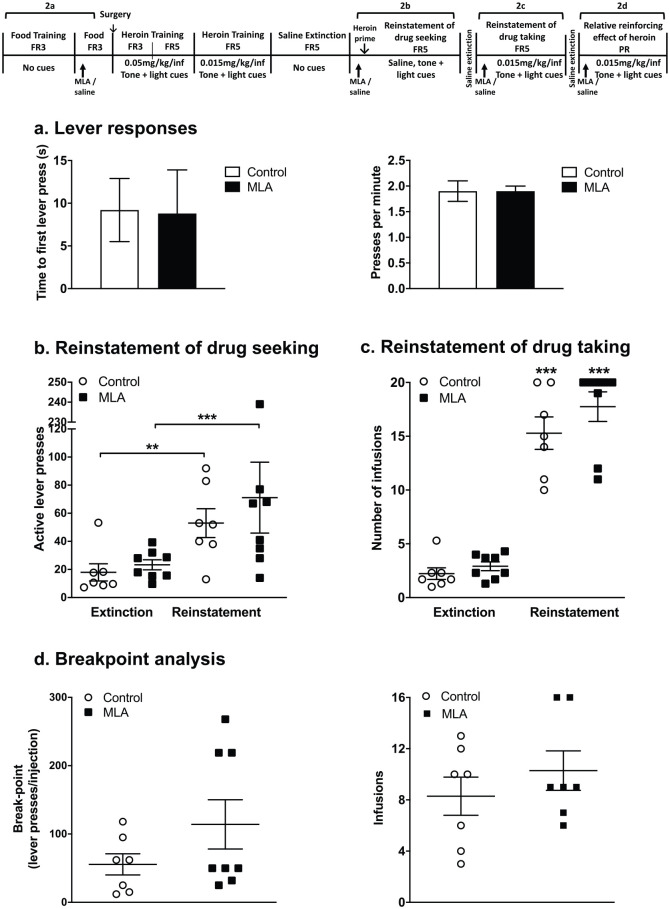
Effect of methyllycaconitine (MLA) on different phases of heroin intravenous self-administration (IVSA). (a) Effect of MLA on active lever responding behaviour (time to first press and presses per minute) for a food reward (fixed ratio (FR)3). (b) Effect of MLA on the heroin- and cue-primed reinstatement of drug-seeking behaviour. Following extinction, rats were pseudo-randomly assigned to two treatment groups and were administered saline (○, 1 mL/kg, s.c. *n* = 7) or MLA (◻, 4 mg/kg, s.c. *n* = 8) 20 min prior to a reinstatement test session, initiated by administration of a priming heroin dose (15 µg/kg, intravenous (i.v.)) and non-contingent presentation of the tone and light cues. Rats received saline infusions paired with the tone and light cues upon the correct number of active lever-presses (FR5). **p = 0.002, ***p < 0.001, multiple *t*-test. (c) Effect of MLA on the reinstatement of heroin IVSA. Rats were administered saline (○, 1 mL/kg, s.c. *n* = 7) or MLA (◻, 4 mg/kg, s.c. *n* = 8) 20 min prior to a reinstatement test session, initiated by a non-contingent heroin infusion (15 µg/kg, i.v.) paired with the tone and light cues. The correct number of active lever-presses (FR5) resulted in heroin injections (15 µg/kg/inj) paired with the tone and light cues. ***p < 0.001, multiple *t*-test. (d) The effect of MLA on the relative reinforcing efficacy of heroin was examined by breakpoint analysis in a single progressive ratio session. After reinstatement as in (c), rats (*n* = 15) remained in their treatment groups and were administered saline (○, 1 mL/kg, s.c.) or MLA (◻, 4 mg/kg, s.c.) 20 min prior to a single progressive ratio session. The session was initiated by a non-contingent injection of heroin (15 µg/kg, i.v.) paired with the tone and light cues. The number of active lever-presses, which resulted in a heroin injection (15 µg/kg/inj) paired with the tone and light cues, was logarithmically increased. Number of lever-presses per infusion (left panel) and number of infusions received (right panel) were recorded.

Twelve of the 26 rats were randomly allocated to either a saline or MLA pre-treatment group in an experiment to assess the effect of MLA on lever-pressing for food rewards on a FR3 reward schedule (Figure 2(a)). Animals were administered either saline (1 mL/kg, s.c.; *n* = 6 rats) or MLA (4 mg/kg, s.c., *n* = 6 rats) 20 min prior to the test session. They were subsequently placed in the operant chambers to lever-press for food pellets on a FR3 schedule for the maximum allotted time of 1 h or until the maximum 50 pellets were dispensed.

### Surgery

After food training and the lever-pressing experiment were completed, rats were anaesthetised by isoflurane (2.5–2.75% in 95% O_2_ and 5% CO_2_) and implanted with chronic indwelling i.v. silicone catheters into the right jugular vein, as described by Smith et al. (2019). Prophylactic antibiotic was administered (baytril 5 mg/kg, s.c.) daily for 48 h, then daily ticarcillin/clavulanic acid (80 mg/kg, i.v.) for the duration of the study. Analgesia was given with carprofen (5 mg/kg s.c.) at least 30 min before animals regained consciousness from the surgery. Catheters were filled with sterile fluid (heparinised saline 30 iU/mL) immediately post-surgery and after every experimental session to maintain catheter patency. Catheter patency was confirmed daily by drawing back to observe freely flowing blood in the catheter line. In three rats, catheter patency failed as detected by visible evidence of leakage, by occlusion of the catheter, by lack of blood drawback and by failure to show immediate sedation upon i.v. injection of propofol (1.625 mg/kg i.v.). These rats were culled by a UK Home Office Schedule 1 procedure. All other animals were allowed to recover for 6–7 days before undergoing training to self-administer heroin.

### Heroin self-administration

Rats were trained to self-administer heroin (starting with 50 µg/kg/injection) in daily two-hour sessions starting on a FR3 schedule. Sessions were initiated by a non-contingent infusion of heroin and simultaneous presentation of a stimulus cue light and a 2.9 kHz tone cue set at 65 dB. The house light was illuminated and the left lever was active. The drug was delivered in a volume of 0.5 mL/kg over a period of 4–5 s depending on the weight of the rat; with a 30-second time-out after each drug infusion, during which time the house light remained on and lever-pressing had no programmed consequences. When rats had approximately eight FR3 sessions, the response requirement was increased to FR5. The final heroin acquisition dose of 15 µg/kg/inj and the FR5 schedule of drug reinforcement were selected based on previous studies using heroin and other opioid reinforcers (Oakley et al., 2015; Smith et al., 2019). Rats were allowed to self-administer a maximum of 20 injections per two-hour session. Rats had at least five sessions on the final heroin dose. Experimental sessions were conducted 6–7 days per week.

### Saline extinction

After the completion of the heroin self-administration training, rats were subjected to extinction sessions. In daily sessions, heroin-maintained lever responding was extinguished by substituting heroin with saline (0.5 mL/kg/inj) and withholding the tone and light cues. Responses on the active lever on the FR5 schedule (30 s timeout) resulted in the delivery of saline. At least five extinction sessions were given after heroin training.

## Effect of MLA on reinstatement of heroin-seeking and heroin self-administration

After completion of the extinction phase, rats were divided into two groups in a pseudo-random manner based on similar levels of active lever-press responding during the acquisition and extinction of heroin self-administration (Group 1 (saline): 171 ± 27 active lever-presses during acquisition and 18 ± 6 at extinction; Group 2 (MLA): 172 ± 22 active lever-presses during acquisition and 21 ± 3 at extinction). Twenty minutes prior to the reinstatement session, rats were administered either saline (1 mL/kg, s.c., *n* = 7) or MLA (4 mg/kg, s.c., *n* = 8).

### Reinstatement of heroin-seeking

The reinstatement of drug-seeking was initiated by a heroin priming dose (15 µg/kg, i.v.) delivered through the indwelling catheter, immediately before the test session. The session was initiated by non-contingent presentation of the tone and light cues. Upon the correct number of active lever-presses (FR5), rats received saline infusions paired with the tone and light cues, with a 30 s timeout, where active lever-presses had no programmed responses. The test session lasted 2 h or until the maximum of 20 injections was achieved. The test session took place once daily on four consecutive days.

### Reinstatement of heroin self-administration

After a second saline extinction phase (two sessions), the reinstatement of heroin self-administration was initiated by the non-contingent presentation of a heroin infusion (15 µg/kg i.v.) and simultaneous presentation of the tone and light cues. Throughout the test session, responses on the active lever on the FR5 schedule resulted in the delivery of heroin (15 µg/kg/inj) paired with the presentation of the tone and light cues. The test session lasted 2 h or until a maximum of 20 injections was achieved. Each rat underwent between 4–6 sessions (once per day), to stabilise responding and the results presented are the mean of the last three sessions.

### Effect of MLA on the relative reinforcing effect of heroin

When stable responding was again achieved on the FR5 schedule, the break-point for responding was determined in a single four-hour progressive ratio (PR) session using a logarithmic PR schedule (Roberts et al., 1989; Table 1). Rats remained in their reinstatement treatment groups and received saline (1 mL/kg, s.c.) or MLA (4 mg/kg, s.c.) 20 min prior to the PR session. The session was initiated by a non-contingent injection of heroin (15 µg/kg, i.v.) paired with the presentation of the tone and light cues. Animals received heroin (15 µg/kg/inj) paired with the presentation of the tone and light cues with each infusion, as the number of required active lever-presses was logarithmically increased. After 2 h, if a period of 30 min elapsed with no drug infusions earned, the session was terminated.

**Table 1.** Progressive ratio schedule.

No of lever-presses	5	7	9	12	15	20	25	32	40	50	62	77	95	118	145	178	219	268	328
Cumulative rewards	1	2	3	4	5	6	7	8	9	10	11	12	13	14	15	16	17	18	19

### Testing criteria

#### Food training

Operant responding was deemed stable under the FR3 schedule when the rats earned ⩾45 food pellets within 1h over four consecutive sessions.

#### Acquisition of heroin IVSA

Positive reinforcement criterion was a mean ⩾12 inj/session over three consecutive sessions. Eight rats did not meet this criterion and were excluded from the study.

#### Extinction

The criterion for extinction for individual rats was defined as when the mean number of active lever-presses over the last three sessions was ⩽30% of the group mean for heroin acquisition.

### Statistical analysis

All variables analysed in the heroin IVSA experiments were found to be positively skewed, so a square root transformation was found to be appropriate, to assume normal distribution and equal variance. For acquisition and reinstatement of heroin self-administration and saline extinction, the data used in the analysis were means of the last three sessions. Results from experiments investigating the reinstatement of heroin-seeking were taken from single sessions. Data were square root transformed and analysed by a mixed linear model with treatment as a fixed factor and animal as a random factor (where treatment is a combination of the self-administration test session and the subcutaneous treatment). Comparisons to saline extinction (same subcutaneous treatment) and to subcutaneous saline (same self-administration test session) were by the multiple *t*-test. This mixed model is appropriate for analysing repeated measures data. The PR breakpoint results were square root transformed and analysed by unpaired *t*-test.

## Results

### CPP

#### Effect of MLA on heroin CPP in rats

In agreement with previous studies (see Bardo et al., 1995), heroin (1 mg/kg) elicited a robust CPP (Figure 1(a)–(c)) that was extinguished following 9 days of saline only treatment (Figure 1(c)). To examine the role of α7 nAChRs in heroin CPP, MLA (4 mg/kg, s.c.) was administered systemically 20 min prior to heroin (acquisition, reinstatement) or prior to testing (expression). The dose of MLA used was based on our previous experience (Wright et al., 2019).

Administration of MLA prior to heroin conditioning had no effect on the acquisition of heroin CPP: both control and MLA-treated rats readily acquired heroin CPP (Figure 1(a)). There was no effect of treatment (two-way ANOVA, *F*_(1,44)_ = 0.03, *p* = 0.86) but a significant effect of test (*F*_(1,44)_ = 10.62, *p* = 0.002). Post-hoc analyses showed significant heroin CPP in both treatment groups (saline + heroin habituation −2.1 ± 28.2 s, post-conditioning 94.4 ± 35.7 s vs MLA + heroin habituation −12.3 ± 14.6 s, post-conditioning 95.4 ± 40.6 s, no effect of treatment *p* = 0.86, *n* = 12).

To examine the effect of α7 nAChR inhibition on the expression of heroin CPP, 12 rats underwent heroin conditioning and were subsequently tested for any preference for the drug-paired chamber (post-conditioning test; test day 9); eight of these animals then underwent a subsequent test, carried out 3 days later, with saline or MLA given 20 min prior to that test (test day 12; Figure 1(b)). In the post-conditioning test (test day 9), rats showed a significant preference for the drug-paired compartment (habituation: 5.6 ± 23.7 s vs 99.5 ± 41.2 s post-conditioning, *p* = 0.032, *n* = 12; paired Student’s t-test). On day 12, MLA had no effect on the expression of CPP (saline 104.0 ± 91.6 s vs MLA 132.5 ± 72.4 s, *p* = 0.93, *n* = 4 per group; unpaired Student’s t-test).

MLA was then tested on the reinstatement of heroin CPP: rats acquired heroin CPP which was then extinguished by repeated saline administration associated with both CPP compartments. MLA or saline was administered 20 min prior to a priming dose of heroin (1 mg/kg, s.c.) or saline. Rats were tested for preference in the CPP apparatus immediately after the administration of the priming dose of heroin. There was a significant effect of treatment during reinstatement (two-way ANOVA, *F*_(3,135)_ = 3.7, *p* = 0.013). Post-hoc analyses showed no preference in the saline control group for either compartment (Saline + Saline, Figure 1(c)). In contrast, heroin priming induced a robust reinstatement of preference for the previously drug-paired compartment (Saline + Heroin, Figure 1(c), *p* < 0.001). Indeed the preference scores after heroin-primed reinstatement were substantially higher than after conditioning, an observation previously seen for morphine-primed CPP (Wright et al., 2019). MLA had no effect on preference in saline-primed rats (MLA + Saline, Figure 1(c)), but MLA given prior to the heroin priming dose abolished any preference for the previously drug-paired compartment (Saline + Heroin – 315.9 ± 43.6 s vs MLA + Heroin −46.8 ± 78.4 s, *p* < 0.001). MLA had no effect on locomotor activity during reinstatement, compared with saline (data not shown). The priming dose of heroin significantly reduced total distance moved (and this was reflected in a reduced number of transitions between compartments). This decrease in distance moved was abolished in animals pre-treated with MLA.

### Intravenous heroin self-administration

#### Effect of MLA on operant responding for food rewards

All rats readily acquired lever-pressing for food rewards. Pre-treatment with MLA (4 mg/kg, s.c.) 20 min before the test session had no effect on lever-pressing for food (Figure 2(a)). There was no significant difference in the time to first active lever-press (saline 9.2 ± 3.7 s versus MLA 8.8 ± 5.1 s, *n* = 6 rats/group; unpaired Student’s *t*-test, *p* = 0.96) or active lever-presses/min (saline 1.9 ± 0.2 versus MLA 1.9 ± 0.1, *n* = 6 rats/group; unpaired Student’s *t*-test, *p* = 0.90).

#### Effect of MLA on heroin-primed + cue-induced reinstatement of drug-seeking

After the acquisition of heroin self-administration with drug-paired cues and extinction of operant responding on saline without drug-paired cues, both saline and MLA-treated groups of rats readily reinstated heroin-seeking when challenged with heroin priming + the presentation of the drug-paired tone + light cues (Figure 2(b)). There was no significant difference in active lever-press responses between treatment groups (saline vs MLA, *p* = 0.6), showing a lack of effect of MLA on the reinstatement of heroin-seeking. Reinstatement of heroin-seeking was repeated over three further daily sessions, and in each session, MLA was without effect (data not shown).

#### Effect of MLA on heroin-primed + cue-induced reinstatement of heroin self-administration

Following acquisition and extinction of heroin self-administration, the control group of rats that were pre-treated with saline readily reinstated heroin self-administration (Figure 2(c)). Pre-treatment with MLA (4 mg/kg s.c.) before each session, had no significant effect on the reinstatement of heroin self-administration (Figure 2(c)).

#### Effect of MLA on the relative reinforcing effect of heroin

The effect of MLA (4 mg/kg s.c.) on the relative reinforcing efficacy of heroin was examined in a single PR session (Figure 2(d)). There was no difference in the breakpoints for drug responding (saline: 48.4 ± 15.2 lever-presses/inj versus MLA: 94.7 ± 32.4 lever/presses/inj; unpaired *t-*test, *p* = 0.2), or the number of infusions self-administered by the rats (saline: 8.3 ± 1.5 inj/session versus MLA: 10.3 ± 1.5 inj/session; unpaired Student’s *t*-test, *p* = 0.37).

## Discussion and conclusions

This study corroborates and extends previous findings that MLA attenuates morphine-primed reinstatement of CPP in mice and rats (Feng et al., 2011; Wright et al., 2019). Here we have demonstrated that MLA selectively abolished reinstatement of CPP for the more reinforcing morphine analogue, heroin, indicating a consistent role for α7 nAChRs in the reinstatement of place preference for opioid drugs. Extending this study to compare the effects of MLA in an operant model using psychologically dependent rats revealed that MLA did not prevent the reinstatement of heroin-seeking initiated by heroin priming + the presentation of drug-paired cues, reinstatement of heroin self-administration or diminish the relative reinforcing effect of heroin experienced by these animals.

To aid the interpretation of the findings from this study, Table 2 describes which aspects of drug liking, reinforcement (abuse) and drug dependence are respectively modelled by CPP, IVSA and reinstatement of drug-seeking. Table 2 identifies the specific points in the transition from drug liking, through escalating drug taking (reinforcement/abuse) leading to psychological dependence and, ultimately, in the case of the opiates to psychological and physical dependence. Since there are multiple definitions for terms like ‘abuse’, ‘dependence’ and ‘addiction’, the ones which we have used have been provided for clarity. Moreover, when investigating the involvement of α7 nAChRs in these phenomena, it is important to appreciate that each pharmacological class of abuse drugs has unique features that create their own cycle of dependence and addiction with key differences in the relative contribution of individual triggers for relapse, and different susceptibilities to pharmacological treatments.

**Table 2.** Animal models commonly used to investigate aspects of drug reward, reinforcement (abuse) and dependence.

Aspect of drug reward/reinforcement/dependence	Animal model
**Model of drug liking (reward)**	
Reward + contextual association	CPP
Passive development of ‘drug-liking’	
**Model of drug abuse (psychological dependence)**	
Reward + contextual association	IVSA meeting the following criteria:
Escalating volitional drug intake	(a) The animals have satisfied the criteria for robust and prolonged drug self-administration (acquisition)
Drug craving	(b) The animals have undergone extinction to eliminate false responders
**Model of psychological and physical dependence**	
Escalating volitional drug intake	IVSA in animals that have either been allowed to self-administer drugs without limits for prolonged periods (e.g. 24 h access for a period of weeks), or IVSA experiments performed in animals that have been rendered physically dependent by administering large doses of drugs to them for prolonged periods.
Drug craving	
Pharmacological tolerance	
Physical dependence on withdrawal	

CPP: conditioned place preference; IVSA: intravenous self-administration.

Drug reward: the psychoactive properties of the substance produce pleasurable experiences that can lead to voluntary escalation of intake and the development of psychological dependence (reinforcement) leading to drug-seeking (craving).

Abuse (psychological dependence): state in which craving for the rewarding effect of the substance produces compulsive and perseverative drug-seeking and taking (reinforcement). In psychological dependence, the balance of motivation is still in favour of the positive effects (reward) over the negative effects (preventing the negative psychological consequences of abstinence (craving)).

Dependence (psychological and physical): escalating substance intake driven by the development of pharmacological tolerance, and in the case of the opiates, physical dependence during periods of withdrawal/abstinence. In substance dependence, the balance of motivation for abuse shifts from positive effects (reward) to negative effects (to prevent the negative consequences of abstinence of psychological dependence (craving) and physical dependence (withdrawal)).

As the name states, CPP is a model of ‘drug liking’. An unconditioned stimulus, e.g. heroin, is paired with a conditioned stimulus (compartment) to form a conditioned response (Aguilar et al., 2009; Tzschentke, 2007). Subjects learn to positively associate the paired CPP chamber (which acquires an incentive value) with the unconditioned stimulus; this is measured as the drug-free preference score for that compartment, taken to be an index of drug liking. In these experiments and in our previous study, rats and mice were subjected to 2 days of exposure to a µ-opioid agonist, i.e. morphine or heroin, enough time for the animals to express a preference in CPP, but insufficient exposure to induce psychological dependence. Distinct learning and memory processes and brain circuitry underpin the establishment of CPP and the retrieval of memories in reinstatement of CPP (Boulton and Moody, 2004). This is highlighted by the selective effect of MLA to abolish heroin-primed reinstatement (Figure 1(c)), but not acquisition or expression, of CPP; these observations are consistent with our previous findings for morphine CPP (Wright et al., 2019). Since MLA is a selective α7 nAChR antagonist, the result implicates a role for nicotinic cholinergic signalling in the retrieval of conditioned memories of drug liking.

**Figure 3.**
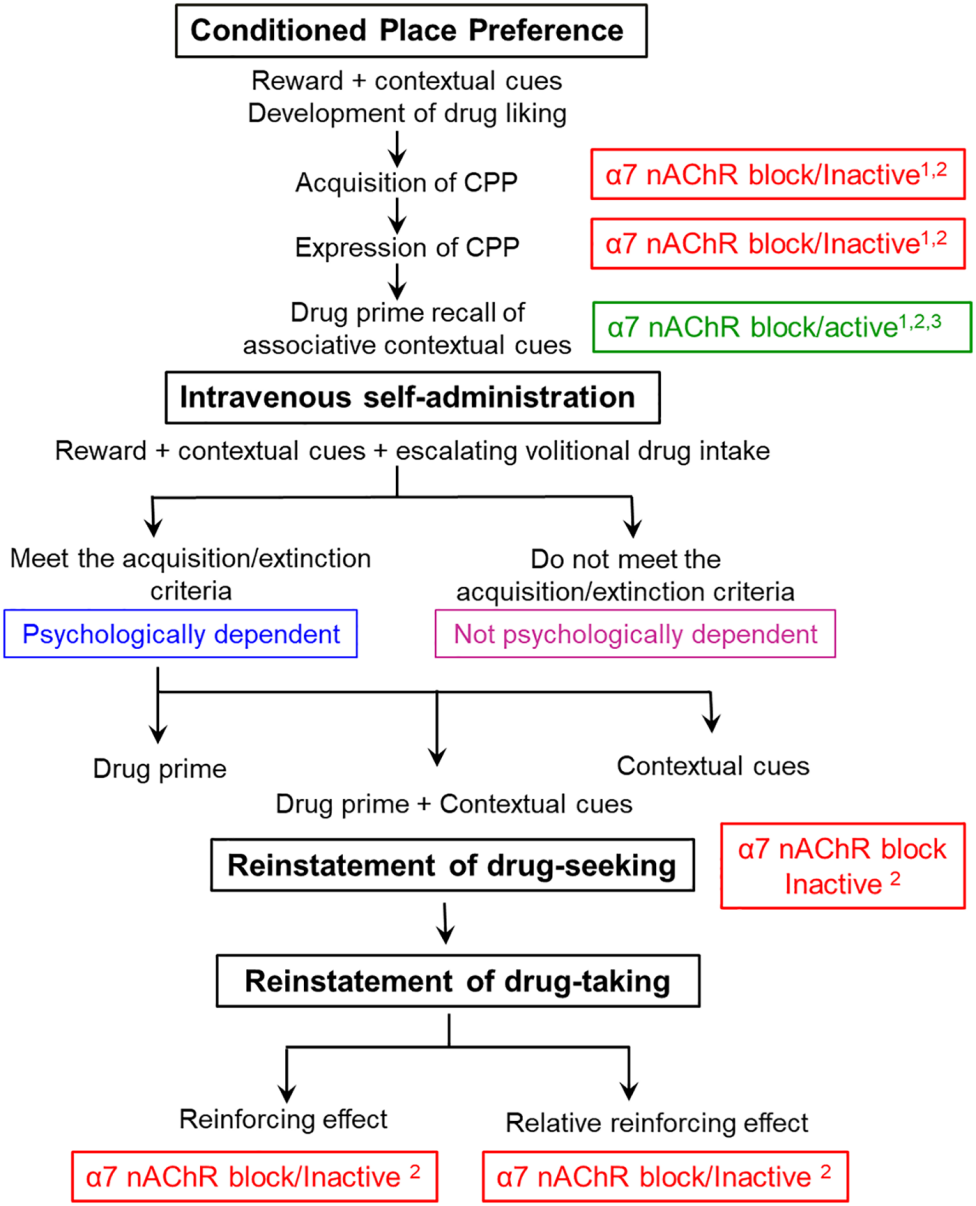
Cascade for the development of opiate liking, psychological dependence, reinstatement of drug-seeking and relapse to opiate abuse showing the aspects where the involvement of α7 nicotinic receptor systems have been investigated. CPP: conditioned place preference; MLA: methyllycaconitine; nAChR: nicotinic acetylcholine receptor. Source: results taken from: Wright et al. (2019),^1^ this study,^2^ Feng et al. (2011).^3^

In contrast, MLA had no effect on any component of heroin self-administration measured. As illustrated in Figure 3, the acquisition of robust heroin self-administration followed by saline extinction models the next phase of the abuse/dependence cascade where animals actively work to obtain access to the drug and, within set limits, can take as much as they desire. In order to study the effects of MLA in the sub-group of rats that were psychologically dependent on heroin, we selected those rats which showed high levels of heroin self-administration and good extinction on saline. The drive for drug-seeking was very powerful in these psychologically dependent rats as shown by the very high number of active non-rewarded lever-presses that they made when reinstatement was initiated by the combination of heroin priming + tone/light cues. Blocking α7 nAChRs did not prevent either their recall of which lever was paired with heroin, or their motivation to seek the drug. Reinstatement of drug-seeking can be elicited by drug-associated cues, drug priming, stress and every combination thereof. In the case of µ-opioid agonists, drug re-exposure (heroin priming) is the major driver to reinstating heroin-seeking with cues having only a minor role (Heal and Smith, 2019). Therefore, it is important to acknowledge that MLA may prevent the reinstatement of heroin-seeking if it had been initiated by cues alone, or even heroin priming on its own. Moreover, when these psychologically dependent rats were given access to heroin, MLA neither reduced the number of infusions that they self-administered, nor decreased heroin’s reinforcing effect. This result mirrors the lack of effect of MLA on the acquisition or expression of µ-opioid agonist-induced CPP (Wright et al., 2019; this study).

As seen in this study and in Table 2, these models reflect different aspects of drug-seeking behaviours. While these paradigms cannot be directly compared, they do offer a certain predictive validity, as conditions that trigger craving and relapse in humans (drug re-exposure, contextual cues and stress) also reinstate drug seeking behaviours in animal models. There are also instances of pharmacological manipulation of the reinstatement of CPP and IVSA which contradict each other, such as dopamine D1 and D2 receptor antagonists, which inhibit the drug-primed reinstatement of heroin IVSA (See, 2009; Shaham and Stewart, 1996), but have no effect on the morphine-primed reinstatement of CPP (Ribeiro Do Couto et al., 2005). The lack of full consensus in the literature on the reinstatement of both paradigms suggests a dissociation of the neurobiological mechanisms mediating these behaviours. Indeed, many reviews have discussed the different brain circuitry recruited during drug-primed, cue-primed or combined drug- and cue-primed reinstatement of drug seeking (Brown and Lawrence, 2009; Namba et al., 2018; Stewart, 2008).

No previous studies have investigated the role of α7 nAChRs in opiate drug-seeking, whereas there is a limited literature for other psychoactive substances. MLA, at comparable doses to that used in the present study, was reported to reduce several behavioural and neurochemical responses to δ-9-tetrahydrocannabinol, including IVSA (Solinas et al., 2007), in contrast to the lack of effect of MLA and α7 nAChRs on nicotine IVSA (Grottick et al., 2000; Pons et al., 2008). However, it has been proposed that blockade of α7 nAChRs prevents the reinstatement of drug-seeking for nicotine (Secci et al., 2017) as well as for cannabinoids (Justinova et al., 2013) and cocaine (Secci et al., 2017), suggesting that α7 nAChRs may serve a common role in the reinstatement of drug-seeking. However, all of these studies relied on modulating levels of kynurenic acid. Although kynurenic acid is a putative α7 nAChR blocker, in addition to its established role as an NMDA receptor antagonist, a recent reappraisal casts doubt on it having direct nicotinic actions (Stone, 2020). Liu (2014) has also reported that MLA inhibited the cue-induced reinstatement of nicotine-seeking; however as there was no evidence to show that the low number of active lever-presses elicited by the nicotine-paired cues was significantly greater than responding in extinction, the results can be discounted as inconclusive. Together with the negative results from the present study, there is little compelling evidence for a specific role for α7 nAChRs in reinstatement of drug seeking monitored by IVSA, in contrast to a robust effect in the reinstatement of CPP.

We previously hypothesised that in CPP, α7 nAChRs play a specific role in the retrieval of drug-associated memories, triggered by cue or drug-prime, that are important in relapse behaviour (Wright et al., 2019). This view is supported by identification of the ventral hippocampus as the locus of inhibition by MLA of morphine-primed reinstatement of CPP (Wright et al., 2019). The ventral hippocampus has been associated with motivation and emotional states of ‘frustration and disappointment’ (Fanselow and Dong, 2010). Its key role in limbic circuitry gives the ventral hippocampus connections to multiple areas (including the nucleus accumbens, amygdala, bed nuclei of the stria terminalis, prefrontal cortex and insular cortex) that would facilitate motivated behaviour such as drug-seeking. For example, glutamatergic projections from the ventral hippocampus to the ventral tegmental area or nucleus accumbens can contribute to cue-induced drug-seeking (Namba et al., 2018; Zhou et al., 2019) and bilateral administration of gamma-aminobutyric acid receptor antagonists into the ventral hippocampus attenuated both cue- and drug-induced reinstatement of drug-seeking in an IVSA model of cocaine addiction (Rogers and See, 2007). The failure of MLA to attenuate reinstatement in two IVSA models of relapse (drug-seeking and drug taking) necessitate re-consideration of a role for α7 nAChRs in reinstatement.

In this study we have identified that the role of α7 nAChRs in the retrieval of drug-associated memories occurs in the initial phase of drug liking before repeated self-exposure to the reinforcing effect of the µ-opioid receptor agonist produces a state of psychological dependence in the animals, suggesting that α7 nAChR antagonists are unlikely to be a viable approach to the treatment of opiate abuse or dependence. The combined findings from the CPP and self-administration models are consistent in showing that blockade of α7 nAChRs does not influence the reinforcing effect of µ-opioid receptor agonists or the development of psychological dependence. The results indicate that the effects of α7 nAChR antagonists on the reinstatement of drug-seeking and relapse into drug abuse are dependent on the point in the drug abuse cascade that is selected for investigation and the trigger(s) used to reinstate drug-seeking.
